# Subcortical motor ischemia can be detected by intraoperative MRI within 1 ​h – A feasibility study

**DOI:** 10.1016/j.bas.2022.100862

**Published:** 2022-01-19

**Authors:** Sebastian Ille, Simon Schoen, Benedikt Wiestler, Bernhard Meyer, Sandro M. Krieg

**Affiliations:** a Department of Neurosurgery, Department of Neurosurgery, Klinikum Rechts der Isar, Technische Universität München, Ismaninger Str. 22, 81675, Munich, Germany; b TUM Neuroimaging Center, Germany; c Department of Diagnostic and Interventional Neuroradiology, Technical University of Munich, School of Medicine, Klinikum Rechts der Isar, Technische Universität München, Ismaninger Str. 22, 81675, Munich, Germany

**Keywords:** Diffusion imaging, Evoked potentials, Ischemia, ioMRI

## Abstract

**Introduction:**

To achieve a maximum extent of resection, intraoperative MRI (ioMRI) scan is frequently performed. Intraoperative diffusion-weighted imaging (DWI) is not standardly performed and has been described to be inferior to early postoperative MRI regarding the detection of ischemia.

**Research question:**

This feasibility study evaluates the detection of ischemia by ioMRI and its clinical relevance in patients with motor-eloquent gliomas.

**Material and methods:**

Of 262 glioma patients, eight patients (3.1%) showed an amplitude loss of continuous motor evoked potential (MEP) monitoring during resection before the ioMRI scan (group loss of MEP ​= ​LOM). In these patients and a matched-pair cohort (MPC) of glioma resections without MEP loss, we performed additional ioMRI sequences including turbo-spin-echo (TSE)- and echo-planar-imaging (EPI)-DWI and perfusion-weighted imaging (PWI). The clinical outcome was measured 5 days and 3 months after surgery.

**Results:**

The mean ​± ​standard time between loss of MEPs and ioMRI was 63.0 ​± ​8.7 ​min (range: 40–84). Ischemia within the motor system could be detected by ioMRI in group LOM in 100% EPI-DWIs, 75% TSE-DWIs, and 66.7% PWIs. No sequence showed motor ischemia in the MPC group. All patients of group LOM and no patient of group MPC suffered from permanent motor deficit.

**Discussion and conclusion:**

The current results provide data on the time sequence of ischemia apparent in MRI sequences which is superior to previous data on symptomatic stroke patients on this topic. The early detection of ischemia adds an additional predictor for the long-term outcome of patients and shows the reason of an intraoperative loss of MEPs. Thereby the performance of intraoperative EPI-DWI might be justified after confirmation of the present data in a larger cohort.

Subcortical ischemia can be detected by ioMRI after MEP loss during the resection of motor-eloquent gliomas and was clinically relevant in all cases.

## Highlights

• Intraoperative MRI was performed after motor evoked potential (MEP) loss in patients with motor-eloquent gliomas.
• Of 262 patients, 8 patients (3.1%) showed an MEP loss during resection.
• Matched-pair cohort (MPC) of glioma resections without MEP loss.
• Mean ​± ​standard time between loss of MEPs and ioMRI was 63.0 ​± ​8.7 ​min (range: 40–84).
• Ischemia within the motor system could be detected by ioMRI in all patients with MEP loss only.

## 1 Introduction

The microsurgical resection of motor eloquent gliomas must avoid surgery-related deficits while achieving a maximum extent of resection (EOR) for an optimal oncological treatment (Stummer et al., 2008; Wijnenga et al., 2018). The gold standard technique for the surveillance of motor function is intraoperative neuromonitoring (IONM) (Krieg et al., 2012; Neuloh et al., 2007; Kombos et al., 2001; Deletis, 1993). Postoperatively, the EOR should be examined within 24–48 ​h and no later than 72 ​h after surgery according to the Response Assessment in Neuro-Oncology (RANO) criteria, not least to receive a baseline image for later adjuvant therapies and follow-up (Wen et al., 2010). Thus, the necessity of a postoperative magnetic resonance imaging (MRI) has repeatedly been reported (Henegar et al., 1996; Ulmer et al., 2006; Smith et al., 2005). Furthermore, particularly in patients with high-grade gliomas, intraoperative MRI (ioMRI) has shown to be beneficial regarding the EOR, the patients’ overall survival and quality of life, at least with level 2 evidence (Kubben et al., 2011; Li et al., 2017; Jenkinson et al., 2018). In contrast, it has been discussed that ioMRI might be inferior to early postoperative MRI regarding the detection of ischemia due to the late appearance of ischemic changes which could be overlooked in diffusion-weighted images (DWI) of ioMRI. Recently, a study compared ischemic lesions as measured by ioMRI and early postoperative MRI within the same scanner in patients who underwent resection of gliomas. The authors came to the conclusion that DWI sequences for the detection of ischemic lesions should only be performed during the early postoperative MRI, since a large proportion of ischemia has been overlooked during ioMRI scans. Most importantly, ischemic lesions were mainly asymptomatic in this publication (Masuda et al., 2018). However, earlier studies have already shown that new postoperative functional deficits are more likely associated with ischemic lesions than with damage of eloquent brain areas (Gempt et al., 2013a, 2013b).

Thus, the present feasibility study aims to evaluate two hypotheses:

1) Subcortical ischemia can be detected within the motor system by ioMRI in patients with a loss of motor evoked potentials (MEP) during the resection of motor-eloquent gliomas.
2) Subcortical ischemia as detected by ioMRI is clinically relevant and predicts the patients' functional long-term outcome.

## 2 Material and methods

### 2.1 Ethics

The study was approved by the local ethics board (Ethikkomission der TU München, Ismaninger Str. 22, 81675 Munich, Germany; registration number: 336/17, 192/18, 18/19). The study was performed in accordance with the Declaration of Helsinki. All included patients provided written informed consent.

### 2.2 Eligibility criteria

We prospectively included patients with suspected cortical or subcortical motor eloquent gliomas as defined by preoperative MRI scan who were scheduled for resection at our department. The indications for tumor resection were made by the interdisciplinary neurooncological board. Patients with an age of less than 18 years or general MRI exclusion criteria were excluded.

### 2.3 Study protocol

#### 2.3.1 MRI scan

We performed a structural MRI scan in all patients (3 ​T MR scanner Achieva, Philips Medical System, Netherlands B.V.) according to the standard MRI protocol including DTI sequences with 32 orthogonal sequences. The same MRI scan was performed postoperatively within 48 ​h after surgery for the final determination of the EOR. A threshold of 5% of residual tumor was defined to separate gross total resection (GTR) and subtotal resection (STR) (Bloch et al., 2012; Southwell et al., 2018).

#### 2.3.2 Intraoperative neuromonitoring

A total intravenous anesthesia (TIVA) was used in all cases. For the transcranial electrical stimulation (TES) for MEP monitoring we used an ISIS stimulator with stimulation needles (inomed Medizintechnik, Emmendingen, Germany) at C3 and C4 as determined by the 20–10 electroencephalography (EEG) system. For direct cortical stimulation (DCS) for MEP monitoring we positioned a strip electrode with 4 contacts over the primary motor cortex and an additional needle electrode at Fpz as the cathodic pole (inomed Medizintechnik, Emmendingen, Germany). The decision on the use of DCS versus TES, and for TES C3–C4 stimulation versus C1–C2 stimulation, was preoperatively made based on the tumor location as shown by MRI, the planned approach to the tumor, cortical versus subcortical eloquence, and cortical or subcortical relevance of IONM in the individual case. Standardly, compound muscle action potentials (CMAP) were recorded at least within three muscles for upper extremity monitoring, and within one muscle for lower extremity monitoring (Krieg et al., 2012). We used a train-of-five stimulation technique. The stimulation parameters were adjusted in case of MEP failure prior to resections and a baseline measurement was performed after the opening of the dura as a reference value for later responses. During the resection MEPs were recorded continuously with intervals of 10 ​s or less. In case of an amplitude decline or complete loss, technical issues or anesthesiological reasons were ruled out first before forwarding to surgeons. After forwarding to the surgeon and prior to the final documentation of an amplitude decline or complete loss, tumor resection was stopped and irrigation with Ringer's solution and vasodilators was performed. Similarly, anesthesiologist were asked for specific events and to optimize parameters such as blood pressure. In case of still persisting MEP changes, an amplitude decline of more than 50% of the baseline amplitude was considered to be significant and was documented as a decline for the present study in case of missing recovery above 50% of the baseline amplitude. Onset of amplitude decline or a complete loss or recovery were documented to the minute. Furthermore, the number of declined or lost MEPs with respect to specific muscles was documented (Krieg et al., 2012; Neuloh et al., 2007; Taniguchi et al., 1993).

#### 2.3.3 Microsurgical tumor resection and intraoperative MRI scan

Microsurgical tumor resections were performed via cavitronic ultrasound aspirator under general anesthesia and by the use of neuronavigation as well as continuous IONM with TES or DCS monitoring (Krieg et al., 2012, 2013).

Our department has a two-room ioMRI setup which is used for all glioma cases in a MRI-compatible headclamp with included coil array (Noras MRI products, Hoechberg, Germany). Preoperatively determined cortical MEP-positive sites by navigated transcranial magnetic stimulation (nTMS) motor mapping and nTMS-based diffusion tensor imaging fiber tracking (DTI FT) of the corticospinal tract (CST) were displayed at the neuronavigation system throughout the whole resection. After the resection was completed, IONM needles were removed for safety reasons prior to ioMRI scanning. The scanner room for the ioMRI was cleaned 40 ​min before the ioMRI scan. Subsequently, ioMRIs were performed according to the standard ioMRI protocol at our department (Dinevski et al., 2017). In short, after the completion of the initial resection and hemostasis, the resection cavity was refilled with ringer's solution and prophylactically closed with a collagen sponge and rough suture. The approach was covered with three sterile layers. The patient was then transferred to the ioMRI scanner after the completion of checklists. This interval for the preparation of the ioMRI was the most time-consuming factor affecting measured durations between the loss of MEPs and ioMRI.

We performed a protocol of sequences including turbo-spin-echo (TSE)- and echo-planar-imaging (EPI)- diffusion-weighted imaging (DWI) with according apparent diffusion coefficient (ADC) maps as well as perfusion-weighted imaging (PWI).

#### 2.3.4 Data analysis

All patients underwent clinical examination including motor testing and documentation according to British Medical Research Council (BMRC) scale (0 ​= ​no contraction, 1 ​= ​flicker or trace of contraction, 2 ​= ​active movement with gravity eliminated, 3 ​= ​active movement against gravity, 4 ​= ​active movement against gravity and resistance, 5 ​= ​normal power) preoperatively, postoperatively, and at 3 months follow-up. Surgical details were documented standardly. MRI scans with regard to EOR and especially with regard to the detection of ischemia were independently rated by at least two board-certified neuroradiologists and two board-certified neurosurgeons. In case of disagreement, a further board-certified neuroradiologist and neurosurgeon was consulted. Ischemic lesions in a thin linear rim around the resection cavity were excluded from the present analysis based on prior publications (Smith et al., 2005).

To evaluate the reliability of detecting ischemia within the motor system by ioMRI and to compare outcome parameters and ioMRI procedures, we performed a matched-pair analysis (group matched-pair cohort ​= ​MPC). Patients without loss or decline of MEPs during IONM who also received the extended ioMRI protocol were matched according to baseline characteristics, tumor location and tumor entity.

Statistical analyses were performed using GraphPad Prism software (GraphPad Prism 8, San Diego, CA, USA). The baseline characteristics of the two groups were compared by independent t-tests and Fisher's exact or chi-square test. A p-value <.05 was considered significant. Initially, Gaussian distribution was tested for all measures. Because of the small cohort size, Gaussian distribution was additionally tested using the Shapiro-Wilk-Test. In case of rejecting the null hypothesis, further calculations for the tested data were performed using the Mann-Whitney Test.

## 3 Results

Between July 2018 and January 2020 we screened 262 patients who underwent microsurgical glioma resection. Of these, eight patients (3.1%; 4 female) with a mean ​± ​standard deviation (SD) age of 52.4 ​± ​16.0 years showed a loss of MEPs as measured by IONM during microsurgical tumor resection (= group loss of MEP ​= ​LOM). These patients were included in the present study and received special sequences for the detection of ischemia during ioMRI according to the study protocol. Additionally, we analyzed a matched-pair cohort (= group matched-pair cohort ​= ​MPC) of eight patients (3.1%; 4 female) with a mean age of 59.1 ​± ​12.8 years of the same cohort, who did not show a loss of MEPs as measured by IONM during microsurgical tumor resection and also received special sequences for the detection of ischemia during ioMRI. Histopathologically, all tumors were classified as gliomas. Table 1 shows detailed patient and tumor characteristics (Table 1).

**Table 1:** Baseline characteristics.

ID	Gender	Age	Diagnosis	WHO	Recurrent tumor	Hemisphere	Lobe
1	M	69	GBM	IV	X	R	F
2	F	41	OD	II	X	L	I
3	M	25	GBM	IV	X	L	T
4	F	67	GBM	IV		L	I
5	M	44	AC	III	X	L	F
6	F	39	GBM	IV		L	F
7	M	65	GBM	IV	X	R	F
8	F	69	GBM	IV		R	I
p	>.9999	.7383	.7333	.7333	>.9999	>.9999	0.8096
9	M	39	AC	III	X	R	I
10	M	81	GBM	IV		L	F
11	F	66	GBM	IV		R	F
12	M	57	GBM	IV		R	I
13	M	43	GBM	IV	X	L	I
14	M	66	GBM	IV	X	R	F
15	F	66	GBM	IV	X	L	F
16	F	55	GBM	IV	X	L	T

The table shows baseline characteristics of all included patients (M ​= ​male, F ​= ​female, GBM ​= ​glioblastoma, OD ​= ​oligodendroglioma, AC ​= ​astrocytoma, WHO = World Health Organization, R ​= ​right, L ​= ​left, F ​= ​frontal, I ​= ​insular, T ​= ​tempora, X ​= ​yes, no entry ​= ​no).

Surveillance of motor function during tumor resection was performed by TES- and DCS-monitoring in six and two cases of group LOM and by TES-monitoring in all cases of group MPC (p ​= ​.2333). The ranges of intensities for TES- and DCS-monitoring were 65–130 ​mA and 5–13 ​mA, respectively. The mean duration of surgery in group LOM was 238.8 ​± ​47.5 (range 159–292) min and 245.3 ​± ​62.2 (range 125–359) min in group MPC (p ​= ​.8295). The mean duration of ioMRIs (time between insertion and removal of collagen sponge) was 68.1 ​± ​2.0 (range 65–70) min in group LOM and 75.4 ​± ​14.0 (range 40–86) min in group MPC (p ​= ​.0095). This discrepancy between the two groups could not be referred to a specific cause. No adverse events occurred in both groups.

**Fig. 1:**
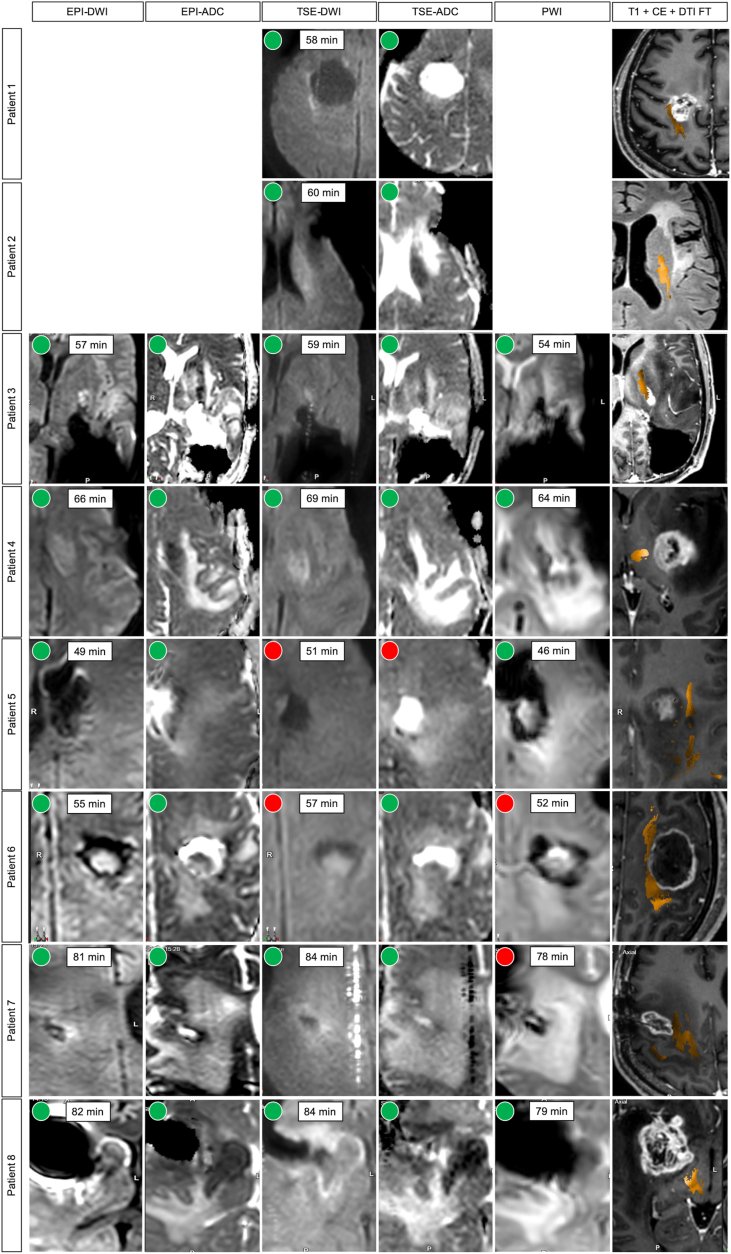
Detection of ischemia within the motor system in ioMRI. The figure shows the diffusion-weighted imaging (DWI) and perfusion-weighted imaging (PWI) sequences as well as the location of the corticospinal tract (orange) as visualized by diffusion tensor imaging fiber tracking (DTI FT) fused with preoperative T1-weighted images with contrast enhancement (T1 ​+ ​CE) of all patients who showed a loss or decline of motor evoked potentials (MEP) during tumor resection. Green circles show that ischemia could be detected by the according sequence. Red circles indicate that ischemia could not be detected. The intervals between the loss or decline of MEPs and the completion of the image is shown for each sequence in minutes (EPI ​= ​echo-planar-imaging, ADC ​= ​apparent diffusion coefficient, TSE ​= ​turbo-spin-echo, PWI ​= ​perfusion-weighted imaging). (For interpretation of the references to colour in this figure legend, the reader is referred to the Web version of this article.)

The mean duration between the loss of MEPs and the first DWI sequence of the ioMRI in group LOM was 63.0 ​± ​13.4 (range 40–84) min (Table 2). In detail, we measured mean intervals starting at the loss of MEPs and the scanning of EPI sequences of 65.0 ​± ​12.7 (range 49–82) min, TSE sequences of 65.3 ​± ​11.8 (range 51–84) min, and PWI sequences of 62.2 ​± ​12.7 (range 46–79) min. The mean duration between the loss of MEPs and the detection of ischemia was 65.0 ​± ​12.7 (range 49–82) min for EPI-DWI and EPI-ADC, 69.0 ​± ​11.2 (range 58–84) min for TSE-DWI, 67.3 ​± ​11.2 (range 57–84) min for TSE-ADC, and 60.8 ​± ​12.3 (range 46–79) min for PWI (Fig. 1).

**Table 2:** Surgical details.

ID		IONM		Duration (min)			EOR	
	Technique	Approach	Event	Surgery	ioMRI	IONM - DWI	ioMRI	2nd resection
1	TES	F	Loss	214	69	58	GTR	–
2	DCS	F-T-P	Loss	292	65	60	GTR	–
3	TES	P–O	Loss	253	67	57	STR	–
4	TES	F-T	Loss	266	70	66	GTR	–
5	TES	F	Loss	159	70	40	GTR	–
6	DCS	F-T-P	Loss	174	69	57	GTR	–
7	TES	F	Decline	289	70	84	STR	GTR
8	TES	F-T	Loss	263	65	82	GTR	–
P	.2333	.6422	<.0001	.8295	.0095	–	>.9999	.7333
9	TES	F-T	No	285	80	–	GTR	–
10	TES	F	No	359	84	–	GTR	–
11	TES	F	No	232	84	–	STR	GTR
12	TES	F-T	No	266	76	–	GTR	–
13	TES	F-T-P	No	229	80	–	STR	STR
14	TES	F	No	253	73	–	GTR	–
15	TES	F	No	125	40	–	GTR	–
16	TES	F-T	No	213	86	–	GTR	–

The table shows surgical details of all included patients (IONM ​= ​intraoperative neuromonitoring, TES ​= ​transcranial electrical stimulation, DCS ​= ​direct cortical stimulation, ioMRI ​= ​intraoperative magnetic resonance imaging, IONM – DWI ​= ​time between loss or decline of motor evoked potentials and start of DWI sequences, EOR ​= ​extent of resection, GTR ​= ​gross total resection, STR ​= ​subtotal resection; decline of IONM was defined as a decrease of >50% of the motor evoked potential amplitude in comparison to baseline). Column 3 describes the surgical approach decisive for the application of TES or DCS and used for tumor resection (F ​= ​frontal, F-T ​= ​fronto-temporal, F-T-P ​= ​fronto-temporo-parietal, P–O ​= ​parieto-occipital).

Loss of MEPs of group LOM correlated with subcortical ischemia within the motor system as measured by the ioMRI in 100%. Single ioMRI sequences correlated with loss of MEPs in 100% (EPI-DWI), 100% (EPI-ADC), 75% (TSE-DWI), 87.5% (TSE-ADC), and 66.7 (PWI) of cases. In the present cohort, we did not find cases with a loss of MEPs and no subcortical ischemia within the motor system. Fig. 1 shows ioMRI sequences of group LOM as well as detailed intervals between the loss of MEPs and single sequences (Fig. 1).

The detection of subcortical ischemia within the motor system by ioMRI correlated with a permanent motor deficit in all patients of group LOM. No sequence showed motor ischemia in the MPC group. No patient of group MPC suffered from permanent motor deficit. Patients of group LOM suffered from slight preoperative motor deficits in 6 cases and in 1 case of group MPC. These did not base on subcortical motor ischemia as controlled by preoperative MRI scan (Table 3).

**Table 3:** Ischemia within the motor system in MRI and functional outcome.

ID	Motor ischemia in ioMRI					PostOPMRI	Course of motor function		
	EPI-DWI	EPI-ADC	TSE-DWI	TSE-ADC	PWI		PreOP	PostOP	Duration
1	N/A	N/A	+	+	N/A	N/A	4	0	Permanent
2	N/A	N/A	+	+	N/A	+	5	0	Permanent
3	+	+	+	+	+	+	4	1	Permanent
4	+	+	+	+	+	+	4	1	Permanent
5	+	+	–	–	+	+	4	0	Permanent
6	+	+	–	+	–	+	4	1	Permanent
7	+	+	+	+	–	+	4	0	Permanent
8	+	+	+	+	+	+	5	0	Permanent
9	–	–	–	–	–	–	5	5	NND
10	–	–	–	–	–	–	5	5	NND
11	–	–	–	–	–	–	4	4	NND
12	–	–	–	–	–	–	5	5	NND
13	–	–	–	–	–	–	5	5	NND
14	–	–	–	–	–	N/A	5	5	NND
15	–	–	–	–	–	–	5	5	NND
16	–	–	–	–	–	–	5	5	NND

The table shows the findings of intraoperative magnetic resonance imaging (ioMRI) and postoperative (PostOP) MRI sequences as well as the long-term outcome of patients as rated by British Medical Research Council scale (0 ​= ​no contraction, 1 ​= ​flicker or trace of contraction, 2 ​= ​active movement with gravity eliminated, 3 ​= ​active movement against gravity, 4 ​= ​active movement against gravity and resistance, 5 ​= ​normal power); N/A ​= ​not available, PreOP ​= ​preoperatively, + ​= ​positive MRI signal, - ​= ​negative MRI signal, NND ​= ​no new surgery-related deficit).

## 4 Discussion

By the present results we were able to show that subcortical ischemia can be detected by ioMRI in patients with a loss of MEPs during the resection of motor-eloquent gliomas at a very early stage. Ischemia within the motor system could be detected in any of the ioMRI sequences in all cases within approximately 1 ​h after the intraoperative loss of MEPs. The shortest durations for the proof of ischemia by ioMRI were 49 ​min for EPI-DWI and EPI-ADC, 58 ​min for TSE-DWI, 57 ​min for TSE-ADC, and 46 ​min for PWI. It must be emphasized that durations presented in this study measure the time between the earliest electrophysiological correlate of an intraoperative ischemic event. The event per se already occurred minutes before the loss of MEPs.

For single ioMRI sequences we found a positive correlation with a loss of MEPs in 100% (EPI-DWI), 100% (EPI-ADC), 75% (TSE-DWI), 87.5% (TSE-ADC), and 66.7% (PWI). Hence, especially standard EPI sequences seem to be qualified for the early detection of ischemia during ioMRI. In contrast, TSE-DWI and PWI sequences which were performed due to the study protocol, did not give additional information for the detection of subcortical motor ischemia. The reliability of the present results could be shown by the results of the MPC group.

Furthermore, subcortical ischemia as detected by ioMRI was clinically relevant and led to a permanent motor deficit in all patients of group LOM. Here, it must be highlighted that the study was focused on patients with an intraoperative loss of MEPs, being the most relevant and strongest predictor of postoperative motor deficits. The detection of subcortical ischemia by ioMRI might show the underlying pathology of an intraoperative loss of MEPs and could have an additional predictive value regarding the long-term outcome of patients. Since ioMRI is usually performed after the resection is preliminarily completed, it neither prevents ischemia-related motor deficits nor substantially changes the surgical strategy. However, by adding an additional predictor for the long-term outcome of patients and in some circumstances showing the reason of an intraoperative loss of MEPs, the results of our study support the performance of at least standard DWI during ioMRI. As shown by the mean durations, the scanning of additional sequences did not result in an extensive prolongation of ioMRI procedures.

Additionally, without taking literature as a basis, it has been discussed that perilesional ischemia visualized in postoperative MRI is based on intermediate-term vasospasm. Based on the present results we could show that these changes are already present during surgery while their pathophysiology remains subject for discussion.

Prior studies have already shown that ischemic lesions as detected by early postoperative MRI, are associated with functional deficits (Gempt et al., 2013a, 2013b; Bette et al., 2016; Jakola et al., 2014). Yet, all these studies defined ‘early’ as 48 or 72 ​h after surgery, but not 60 ​min after the electrophysiological correlate of an ischemic event.

In contrast to the present results, it has been discussed by others that ioMRI might be inferior to early postoperative MRI regarding the detection of ischemia due to the late appearance of ischemic changes which could be overlooked in DWI of ioMRI. Yet, whether such ischemia happened during resection or due to borderline perfusion and vascular changes afterwards cannot be said for sure, thus, resulting in inadequate or inaccurate results or interpretation of those.

Recently, a study compared ischemic lesions as measured by ioMRI and early postoperative MRI within the same scanner after glioma resection. The authors came to the conclusion that DWI sequences for the detection of ischemic lesions should only be performed during the early postoperative MRI, since a large proportion of ischemic tissue has been overlooked during ioMRI scans. Most importantly, ischemic lesions were mainly asymptomatic in this publication and therefore their accurate time of onset impossible to define (Masuda et al., 2018). Ischemic lesions on standard DWI could be detected in five and 16 of 30 patients by ioMRI and early postoperative MRI in this study. Only three of the 16 ischemic lesions in early postoperative MRI were symptomatic, and the authors did not publish if these have already been detected on ioMRI or detailed information on the functional status of patients and the long-term outcome. The masking of ischemic lesions in ioMRI was reasoned by susceptibility artifacts from the air in the resection cavity in six of eleven patients and by equivocal signal changes in five of eleven patients. Apart from the fact that the whole study cohort was reviewed in this study without relation to IONM results, regarding artifacts, the refilling of the resection cavity with ringer's solution according to our standard ioMRI protocol might have been a reason for a better image quality in our cohort. Moreover, it must be emphasized that we performed ioMRIs using a 3 ​T scanner while a 1.5 ​T scanner was used for the study of Masuda et al. (2018).

What is more, the results of the present study accompany those of earlier trials in stroke patients. It is known that DWI imaging detects ischemia even at very early stages after the event as shown by the imaging of hyperacute strokes (Lansberg et al., 2000; Warach et al., 1992; Petkova et al., 2010). Hence, DWI imaging combined with further sequences is used to determine the age of the ischemic lesion (Petkova et al., 2010; Thomalla et al., 2011, 2018). Furthermore, DWI and PWI sequences have been used to predict the outcome after ischemic stroke (Barber et al., 1998). However, CT imaging instead of MRI imaging is still commonly used to detect acute ischemic stroke (Vilela and Rowley, 2017). It is suspected that the detection of very early ischemia cannot be visualized by MRI. Furthermore, studies on stroke patients have to rely on the reported onset of symptoms. In contrast, this could be measured in our study by an approved electrophysiological method to the minute. Thus, our results give detailed information on the time span between deterioration of motor function as measured by IONM and the visualization by MRI in patients for the first time. By the present feasibility study's results we can confirm that ischemia can be visualized by MRI even within approximately 1 ​h after the ischemic event. The mean time between the loss of MEPs and the scanning by ioMRI was 65.0 ​min for EPI sequences, 65.3 ​min for TSE sequences, and 62.2 ​min for PWI sequences. Thereby, the present results might also have a major impact on procedures in acute stroke patients.

The small sample size is a major limitation of our study. On the one hand, this is reasoned by the exclusive inclusion of patients with a loss of MEPs during tumor resection. However, the study cohort as well as the matched-pair cohort are highly homogeneous with regard to patient and tumor characteristics (Table 1). In contrast, more patients of the group LOM already suffered from slight preoperative motor deficits as compared to the preoperative functional status of the MPC group. This fact might have influenced the intraoperative loss of MEPs in the LOM group per se. However, the difference of preoperative status’ between the two groups should not have affected the core results of the present study. Additionally, the presence of preoperative subcortical motor ischemia could be ruled out for both groups by preoperative MRI scans. Furthermore, we found positive correlations and consistent results throughout the entire study cohort. Nevertheless, it must be highlighted that the results of the present study have to be confirmed in a larger cohort in order to fully prove the hypotheses.

From an IONM perspective, the applied techniques TES versus DCS as well as the application mode of TES is a subject for debate. The choice of IONM techniques was individually based on the tumor location and the approach for resection (Table 2). DCS was used in case of a craniotomy with safe access to the central region. Regarding the use of TES, it must be highlighted that the application of C3–C4 versus C1–C2 is risky and has the potential to provide false-negative results in case of CST stimulation distal of the tumor. By the intraoperative course of MEPs and the postoperative outcome we can rule out false-negatives and a CST activation distal of the tumor in the present cohort. However, risks, advantages, and disadvantages of applied IONM techniques must also be considered when discussing the present results.

## 5 Conclusion

Subcortical ischemia can be detected by ioMRI in patients with a loss of MEPs during the resection of motor-eloquent gliomas even 60 ​min after the earliest intraoperative electrophysiological correlate of an ischemic event which was clinically relevant in all cases. Standard EPI-DWI sequences were the most sensitive modality. Due to the select choice of inclusion criteria the sample size of the presented cohort showing an intraoperative loss of MEPs is small. Apart from the clinical perspective with adding an additional predictor for the long-term outcome of patients and in some circumstances showing the reason of an intraoperative loss of MEPs as well as the support of performing intraoperative DWI, the results of the present study should also be rated by their scientific value with showing the very early occurrence and detection of subcortical ischemia. With this in mind, these findings are also of interest in early diagnostics of ischemic stroke.

## Additional information

The authors declare no competing interests.

This research did not receive any specific grant from funding agencies in the public, commercial, or not-for-profit sectors. This trial was funded entirely by institutional grants from the Department of Neurosurgery, Technical University of Munich, Germany, School of Medicine, Klinikum rechts der Isar.

BM received honoraria, consulting fees, and research grants from Medtronic (Meerbusch, Germany), Icotec AG (Altstätten, Switzerland), and Relievant Medsystems Inc., (Sunnyvale, CA, USA), honoraria, and research grants from Ulrich Medical (Ulm, Germany), honoraria and consulting fees from Spineart Deutschland GmbH (Frankfurt, Germany) and DePuy Synthes (West Chester, PA, USA), and royalties from Spineart Deutschland GmbH (Frankfurt, Germany). SK is consultant for Nexstim Plc (Helsinki, Finland) and Spineart Deutschland GmbH (Frankfurt, Germany), and received honoraria from Medtronic (Meerbusch, Germany) and Carl Zeiss Meditec (Oberkochen, Germany). SK and BM received research grants and are consultants for Brainlab AG (Munich, Germany). SI is consultant for Brainlab AG (Munich, Germany). BW and SS do not have anything to disclose.

## Declaration of competing interest

The authors declare the following financial interests/personal relationships which may be considered as potential competing interests:

Sandro M Krieg reports a relationship with Brainlab AG that includes: consulting or advisory. Sandro M Krieg reports a relationship with ulrich GmbH und Co KG that includes: consulting or advisory. Sandro M Krieg reports a relationship with Carl Zeiss Meditec AG that includes: speaking and lecture fees. Sandro M Krieg reports a relationship with Nexstim Oy that includes: speaking and lecture fees. Sebastian Ille reports a relationship with Brainlab AG that includes: consulting or advisory.

## Data Availability

The datasets generated during and analyzed during the current study are available from the corresponding author on reasonable request.

## References

[bib1] Barber P.A. (1998). Prediction of stroke outcome with echoplanar perfusion- and diffusion-weighted MRI. Neurology.

[bib2] Bette S. (2016). Infarct volume after glioblastoma surgery as an independent prognostic factor. Oncotarget.

[bib3] Bloch O. (2012). Impact of extent of resection for recurrent glioblastoma on overall survival: clinical article. J. Neurosurg..

[bib4] Deletis V. (1993). Intraoperative monitoring of the functional integrity of the motor pathways. Adv. Neurol..

[bib5] Dinevski N. (2017). Postoperative neurosurgical infection rates after shared-resource intraoperative magnetic resonance imaging: a single-center experience with 195 cases. World Neurosurg.

[bib6] Gempt J. (2013). Postoperative ischemic changes following resection of newly diagnosed and recurrent gliomas and their clinical relevance. J. Neurosurg..

[bib7] Gempt J. (2013). Postoperative ischemic changes after glioma resection identified by diffusion-weighted magnetic resonance imaging and their association with intraoperative motor evoked potentials. J. Neurosurg..

[bib8] Henegar M.M., Moran C.J., Silbergeld D.L. (1996). Early postoperative magnetic resonance imaging following nonneoplastic cortical resection. J. Neurosurg..

[bib9] Jakola A.S. (2014). Surgically acquired deficits and diffusion weighted MRI changes after glioma resection--a matched case-control study with blinded neuroradiological assessment. PLoS One.

[bib10] Jenkinson M.D. (2018). Intraoperative imaging technology to maximise extent of resection for glioma. Cochrane Database Syst. Rev..

[bib11] Kombos T., Suess O., Ciklatekerlio O., Brock M. (2001). Monitoring of intraoperative motor evoked potentials to increase the safety of surgery in and around the motor cortex. J. Neurosurg..

[bib12] Krieg S.M. (2012). Predictive value and safety of intraoperative neurophysiological monitoring with motor evoked potentials in glioma surgery. Neurosurgery.

[bib13] Krieg S.M. (2013). Reliability of intraoperative neurophysiological monitoring using motor evoked potentials during resection of metastases in motor-eloquent brain regions: clinical article. J. Neurosurg..

[bib14] Kubben P.L. (2011). Intraoperative MRI-guided resection of glioblastoma multiforme: a systematic review. Lancet Oncol..

[bib15] Lansberg M.G. (2000). Advantages of adding diffusion-weighted magnetic resonance imaging to conventional magnetic resonance imaging for evaluating acute stroke. Arch. Neurol..

[bib16] Li P., Qian R., Niu C., Fu X. (2017). Impact of intraoperative MRI-guided resection on resection and survival in patient with gliomas: a meta-analysis. Curr. Med. Res. Opin..

[bib17] Masuda Y. (2018). Evaluation of the extent of resection and detection of ischemic lesions with intraoperative MRI in glioma surgery: is intraoperative MRI superior to early postoperative MRI?. J. Neurosurg..

[bib18] Neuloh G., Pechstein U., Cedzich C., Schramm J. (2007). Motor evoked potential monitoring with supratentorial surgery. Neurosurgery.

[bib19] Petkova M. (2010). MR imaging helps predict time from symptom onset in patients with acute stroke: implications for patients with unknown onset time. Radiology.

[bib20] Smith J.S. (2005). Serial diffusion-weighted magnetic resonance imaging in cases of glioma: distinguishing tumor recurrence from postresection injury. J. Neurosurg..

[bib21] Southwell D.G. (2018). Resection of gliomas deemed inoperable by neurosurgeons based on preoperative imaging studies. J. Neurosurg..

[bib22] Stummer W. (2008). Extent of resection and survival in glioblastoma multiforme: identification of and adjustment for bias. Neurosurgery.

[bib23] Taniguchi M., Cedzich C., Schramm J. (1993). Modification of cortical stimulation for motor evoked potentials under general anesthesia: technical description. Neurosurgery.

[bib24] Thomalla G. (2011). DWI-FLAIR mismatch for the identification of patients with acute ischaemic stroke within 4.5 h of symptom onset (PRE-FLAIR): a multicentre observational study. Lancet Neurol..

[bib25] Thomalla G. (2018). MRI-guided thrombolysis for stroke with unknown time of onset. N. Engl. J. Med..

[bib26] Ulmer S. (2006). Clinical and radiographic features of peritumoral infarction following resection of glioblastoma. Neurology.

[bib27] Vilela P., Rowley H.A. (2017). Brain ischemia: CT and MRI techniques in acute ischemic stroke. Eur. J. Radiol..

[bib28] Warach S., Chien D., Li W., Ronthal M., Edelman R.R. (1992). Fast magnetic resonance diffusion-weighted imaging of acute human stroke. Neurology.

[bib29] Wen P.Y. (2010). Updated response assessment criteria for high-grade gliomas: response assessment in neuro-oncology working group. J. Clin. Oncol..

[bib30] Wijnenga M.M.J. (2018). The impact of surgery in molecularly defined low-grade glioma: an integrated clinical, radiological, and molecular analysis. Neuro Oncol..

